# Epigenetic Mechanisms as Emerging Therapeutic Targets and Microfluidic Chips Application in Pulmonary Arterial Hypertension

**DOI:** 10.3390/biomedicines10010170

**Published:** 2022-01-13

**Authors:** Linh Ho, Nazir Hossen, Trieu Nguyen, Au Vo, Fakhrul Ahsan

**Affiliations:** 1 Department of Pharmaceutical and Biomedical Sciences, College of Pharmacy, California Northstate University, Elk Grove, CA 95757, USA; nazir.hossen@cnsu.edu (N.H.); trieu.nguyen@cnsu.edu (T.N.); 2 East Bay Institute for Research & Education (EBIRE), Mather, CA 95655, USA; 3 Department of Life Sciences, University of California Los Angeles, Los Angeles, CA 90095, USA; voau03@g.ucla.edu

**Keywords:** epigenetics, activators, inhibitors, drug targets, vascular remodeling, RV failure, pulmonary arterial hypertension, PAH, sirtuins, microchips, PAH chips

## Abstract

Pulmonary arterial hypertension (PAH) is a disease that progress over time and is defined as an increase in pulmonary arterial pressure and pulmonary vascular resistance that frequently leads to right-ventricular (RV) failure and death. Epigenetic modifications comprising DNA methylation, histone remodeling, and noncoding RNAs (ncRNAs) have been established to govern chromatin structure and transcriptional responses in various cell types during disease development. However, dysregulation of these epigenetic mechanisms has not yet been explored in detail in the pathology of pulmonary arterial hypertension and its progression with vascular remodeling and right-heart failure (RHF). Targeting epigenetic regulators including histone methylation, acetylation, or miRNAs offers many possible candidates for drug discovery and will no doubt be a tempting area to explore for PAH therapies. This review focuses on studies in epigenetic mechanisms including the writers, the readers, and the erasers of epigenetic marks and targeting epigenetic regulators or modifiers for treatment of PAH and its complications described as RHF. Data analyses from experimental cell models and animal induced PAH models have demonstrated that significant changes in the expression levels of multiple epigenetics modifiers such as HDMs, HDACs, sirtuins (Sirt1 and Sirt3), and BRD4 correlate strongly with proliferation, apoptosis, inflammation, and fibrosis linked to the pathological vascular remodeling during PAH development. The reversible characteristics of protein methylation and acetylation can be applied for exploring small-molecule modulators such as valproic acid (HDAC inhibitor) or resveratrol (Sirt1 activator) in different preclinical models for treatment of diseases including PAH and RHF. This review also presents to the readers the application of microfluidic devices to study sex differences in PAH pathophysiology, as well as for epigenetic analysis.

## 1. Introduction: Definition, Epidemiology, and Clinical Symptoms of PAH

Pulmonary arterial hypertension (PAH) or group 1 pulmonary hypertension (PH) is a disease characterized by an increase in pulmonary arterial pressure and pulmonary vascular resistance that often leads to right-sided heart failure and death. It is defined as a mean pulmonary artery pressure (mPAP) more than or equal to 25 mm Hg at rest, with a pulmonary capillary wedge pressure or left-ventricular end-diastolic pressure (LVEDP) ≤ 15 mm Hg and a pulmonary vascular resistance (PVR) > 3 Wood units (WU) measured by cardiac catheterization [1,2]. 

In a study in the US, the PAH prevalence was 109 cases per million for population who use private insurance companies and 451 cases per million for the Medicare population [3]. However, the PAH prevalence in Europe was reported to range from 15–52 per million [3]; Scottish prevalence was 26 cases per million population [4], and the French PAH registry revealed 26 cases per million individuals [5,6]. This discrepancy might be attributed to differences in the prevalence of associated diseases with PAH among these countries and other variations including period and geographic area, genetic background, and pharmacologic treatment availability [7]. In another study in European countries, the predicted 5-year survival rate was from 32% to 76% depending on severity of the disease identified by risk stratification strategy [8]. The median survival of PAH subjects was estimated to be 2.8 years without an effective treatment after diagnosis [9]. 

The World Health Organization (WHO) has divided pulmonary hypertension into five groups depending on its causes [10].

(1) Pulmonary arterial hypertension (PAH) or Group I
  i. Idiopathic PAH (IPAH: primary pulmonary hypertension) or inherited PAH
  ii. PAH induced by drug or toxins
  iii. Associated PAH (APAH): PAH associated with connective tissue and heart disorders, infection of human immunodeficiency virus portal hypertension, congenital tissue and heart diseases, schistosomiasis, chronic hemolytic anemia, pulmonary capillary hemangiomatosis, pulmonary veno-occlusive disease, and newborn PH
(2) PH associated with left-heart disease (venous PH) or group II (ex. mitral valve)
(3) PH associated with lung disease (hypoxic PH) or group III (ex. COPD, interstitial lung disease, sleep apnea)
(4) PH associated with thromboembolic diseases (thromboembolic PH) or group IV
(5) PH with multifactorial mechanisms (miscellaneous PH) or group V (ex. increased red blood cell production, metabolic abnormalities)

Symptoms of pulmonary hypertension include exercise intolerance, dyspnea, fainting, chest pain, and syncope, eventually resulting in right-ventricular hypertrophy (RVH) and right-ventricular failure (RVF) [6,11,12]. If left untreated, the disease imposes a high rate of mortality, with decompensated right-heart failure leading to death as the most frequent reason [6,11,12]. 

**Figure 1:**
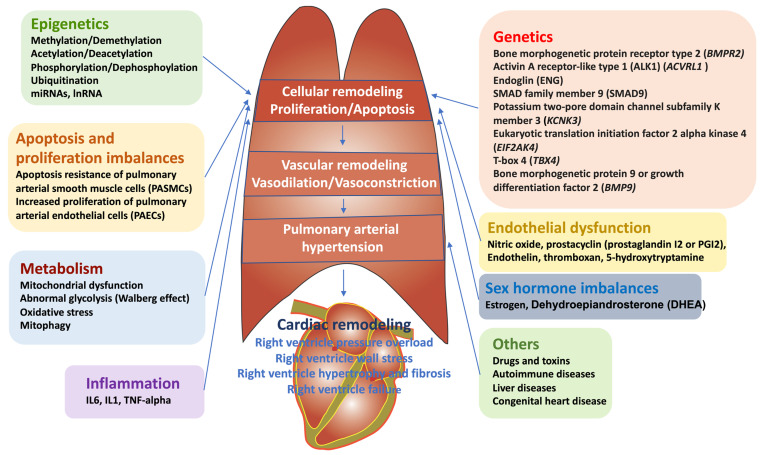
Major pathophysiological mechanisms that lead to vascular remodeling increased pulmonary artery and, thus, right-ventricle remodeling. The sequential pathological events of vascular remodeling in PAH include increased proliferation of smooth muscle cells, subsequent muscularization of peripheral pulmonary arteries, and medial hypertrophy in pulmonary muscular arteries. Further intimal fibrosis occurs by infiltration of inflammatory cells and progressive migration of smooth muscle cells. As a consequence, plexiform lesions and vessel occlusion cause reduced blood flow, resulting in PAH as progression of PAH.

The pathology of PAH is coordinated with many molecular mechanisms including genetic and epigenetic dysregulation, apoptosis resistance and dysfunction of pulmonary arterial endothelial cells (PAECs), increased proliferation of pulmonary arterial smooth muscle cells (PASMCs), inflammation, DNA damage, metabolic diseases, sex hormone disorders, and oxidative stress [11] (Figure 1).

## 2. Current Therapeutic Targets for PAH

PAH is a life-threatening disorder characterized by vascular proliferation and remodeling of small pulmonary arteries [12,13,14]. PAH is defined as dysfunction of pulmonary vascular endothelial and smooth muscle cells, as well as their interaction, resulting from an imbalance in vasoconstriction and vasodilation mediators, as well as surrounding adventitial expansion [12,13,14,15]. Endothelial dysfunction is believed to play an integral role in PAH pathogenesis by decreased production of vasodilators (nitric oxide and prostacyclin) and increased production of vasoconstrictors (thromboxane and endothelin-1) [14].

**Figure 2:**
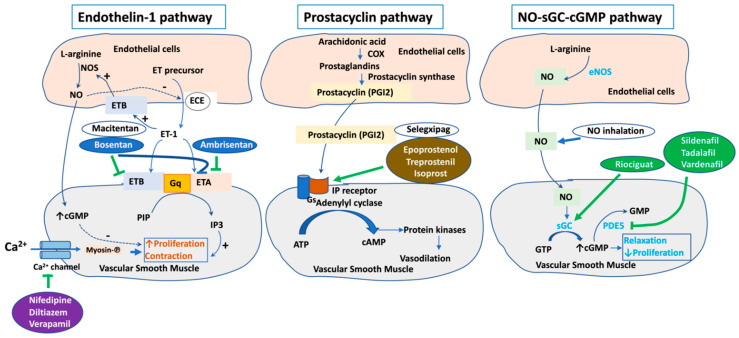
Current therapeutic target pathways for PAH. The endothelin-1, prostacyclin, and nitric oxide pathways have been exploited in clinical therapeutics. These three pathways are critical pathways approved for PAH treatment. Abbreviations: NO, nitric oxide; NOS, nitric oxide synthase; eNOS, enthothelial nitric oxide synthase; GC, guanylyl cyclase; PDE5, phosphodiesterase type 5; GMP, guanosine monophosphate; cGMP, cyclic guanosine monophosphate; GTP, guanosine triphosphate; ET-1, endothelin-1; ETA, endothelin A; ETB, endothelin type A; ECE, endothelin converting enzyme; PIP, phosphatidylinositol phosphate; IP3, inositol 1,4,5-trisphosphate; COX, cyclooxygenase; ATP, adenosine triphosphate; cAMP, cyclic adenosine monophosphate.

In addition, the interaction between dysfunctional endothelial cells and smooth muscle cells enhances 5-hydroxytryptamine (5-HT) production, transport, and activity causing vasoconstriction [16,17]. The net effects of the endothelial dysfunction are vasoconstriction and thickening of the pulmonary arteries, which lead to an elevation in pulmonary vascular resistance and, thus, elevated afterload of the right ventricle that may eventually result in its failure [13,14]. These vasoconstriction and vasodilation factors have been exploited to develop current pharmacologic treatments for PAH. They are antagonists of vasoconstriction factors and agonists of vasodilation factors including endothelin receptor antagonists (ERAs), prostacyclin analogs (PCAs), phosphodiesterase 5 inhibitors (PDE5-Is), and soluble guanylate cyclase stimulators (sGCs) approved by the US Food and Drug Administration (FDA) for PAH treatment [18] (Figure 2). 

Calcium channel blockers (CCBs) such as verapamil (Calan), diltiazem (Cardizem), amlodipine (Norvasc), and nifedipine (Adalat) have also been used for treatment of PAH patients from 1980s [19,20,21,22]. A small number of PAH patients responsive to acute vasodilator testing might be initiated on CCB therapy. CCBs should not be used without demonstrated acute vasoreactivity. Dihydropyridine CCBs (amlodipine, nifedipine) are preferred as they lack the negative inotropic effects seen with verapamil. The most common adverse effect is peripheral edema [23]. 

Soluble guanylate cyclase stimulators such as riociguat work synergistically with nitric oxide and directly activate soluble guanylate cyclase for their pulmonary vasodilation effect [24,25]. Side-effects include headache, dyspepsia, dizziness. It is a teratogenic and, hence, should not be used for pregnant women [23]. 

In addition to riociguat, nitrovasodilators or inhaled nitric oxide (NO) modulating the NO-cGMP signaling pathway has been used for treatment of PAH patients. NO stimulates the formation of cGMP (cyclic guanosine monophosphate) via stimulating soluble guanylyl cyclase (sGC). cGMP in turn reduces intracellular calcium level, resulting in smooth muscle relaxation [26].

Furthermore, phosphodiesterase inhibitors including sildenafil (Revatio), vardenafil (Levitra), and tadalafil (Adcirca), also acting on the NO-cGMP signaling pathway as pulmonary vasodilators, have also been applied for patients with PAH [26]. Phosphodiesterase-5 (PDE5) catalyzes the conversion of cGMP to GMP. cGMP relaxes the smooth muscle cell by decreasing intracellular calcium level. Therefore, by inhibiting PDE5, sildenafil, vardenafil, and tadalafil increase the availability of cGMP, resulting in efficient vasodilation. Side-effects of phosphodiesterase inhibitors are headache, flushing, dyspepsia, and visual disturbances [23]. 

It has been found that prostacyclin (PGI2) synthase decreases in idiopathic PAH patients, associated with reduced PIG2 or prostacyclin release and increased thromboxane-α2 [27,28]. Of note, prostacyclin binds to the prostacyclin receptor in smooth muscle cells and enhances the Gs adenylyl cyclase pathway to increase cAMP (cyclic adenosine monophosphate) level. Through the cAMP-dependent protein kinase A (PKA) pathway, prostacyclin causes smooth muscle cell relaxation (vasodilation). Prostacyclin is also an inhibitor of platelet activation. Therefore, PGI2 and its analogs, as well as prostacyclin IP receptor agonist, have been supplemented in various pharmaceutic formulations to induce vasodilation for treatment of PAH [29,30]. The currently available prostacyclin analogs are epoprostenol (Flolan), iloprost (Ventavis), treprostinil (Remodulin), and selexipag (Uptravi) as an agonist of I-prostanoid receptor [29]. Epoprostenol (Flolan) acts as a vasodilator, as well as an inhibitor of platelet activation and smooth muscle cell proliferation [23,31]. It possesses a short half-life, requiring it to be delivered by continuous intravenous infusion. Therefore, sepsis is a complication that needs to be considered when using this drug. The inhaled prostacyclin analog, iloprost (Ventavis), is also available, but it needs to be given 6–9 times daily. Treprostinil (Remodulin) is a stable PGI2 analog used under subcutaneous (SC) or IV infusion, inhalation, or oral sustained release (longer half-life compared to epoprostenol). This drug has side-effects including headache, flushing, diarrhea, nausea, and jaw pain [23,29].

Endothelin-1 is a vasoconstrictor and stimulator of vascular smooth muscle proliferation secreted from pulmonary endothelial cells (PAECs) in PAH [14]. It is converted from its larger precursor by endothelin converting enzyme (ECE). Endothelin-1 binds both ETA and ETB receptors. Binding to these receptors results in the activation of the Gq–PLC–IP3 pathway and the formation of IP3 (inositol triphosphate). An increase in IP3 is associated with an increase in intracellular calcium level and contraction of smooth muscle. Endothelin-1 might also incorporate to Gi to inhibit cyclic AMP (cAMP) production leading to vasoconstriction [32]. Endothelin receptor antagonists (ERAs) including bosentan (Tracleer), ambrisentan (Letairis), and macitentan (Opsumit) improve cardiopulmonary hemodynamics, exercise tolerance (6 min walk distance), and other symptoms for patients of PAH [33,34,35]. Bosentan inhibits the vasoconstriction and smooth muscle proliferation effects of endothelin. It is an antagonist of both ETA and ETB receptors. Adverse effects are anemia, abnormal liver function tests, peripheral edema, headaches, and nasal congestion. Monitoring liver aminotransferases should be performed monthly [23,30,35]. Ambrisentan (Letairis) selectively antagonizes the ETA receptor used once daily for improving exercise tolerance and hemodynamics, as well as slows down clinical worsening in PAH. Common side-effects include peripheral edema, nasal congestion, flushing, anemia, and palpitations. Unlike bosentan, liver toxicity is rare with ambrisentan [20,25,32,35]. Macitentan (Opsumit) is a once-daily latest dual ERA that acts on both ETA and ETB receptors [25,32,36]. Its side-effects include anemia, headache, and nasopharyngitis. All ERAs are teratogenic and, thus, should not be used in pregnant patients [23].

## 3. Sex Disparity in PAH and Microfluidic Chip Model Mimicking PAH-Insulted Artery to Study Sex Discrepancy in PAH Pathophysiology

The sex disparity affects prevalence, pathogenesis, and treatment response in PAH. PAH cases are more predominant with the female than male population [36,37]. Although PAH is prominent in females, studies have demonstrated that the survival rate is higher in female compared to male patients, also known as the “sex paradox” [38,39,40]. In fact, female patients have shown higher exercise capacity in 6 min walk distance practice and exhibited greater cardiac output that significantly correlated with increased survival compared to male ones [38,39,40]. Moreover, growing evidence suggests that females have a higher right-ventricular ejection fraction at baseline, in addition to being more responsive to drug treatment with a higher tolerance to stress on the heart tissue compared to males despite higher pulmonary afterload as an explanation for increased survival rate [37,41,42,43]. The prominence toward the female sex in PAH has been attributed to the sex hormone discrepancy as contributors to the pathogenic mechanism of the disease, especially estrogen and estrogen metabolites [44,45,46,47]. Furthermore, it has been reported that increased estradiol (E2) was related to a high risk of PAH and a decrease in exercise tolerance in a 6 min walk distance measurement of male PAH patients [48]. This study also showed that increased dehydroepiandrosterone sulfate (DHEA-S) was linked with a low risk of PAH and reduced right-atrial heart pressure and pulmonary vascular resistance in PAH males [48]. Taken together, further studies are in need to elucidate the role of other sex hormones and their metabolites, as well as sex hormone signaling and synthesis in pathogenesis, prevalence, and outcome of PAH. 

The sex paradox in PAH can be explained by the notion that women have a more fit and responsive immune system than men because of their partial inactive additional X chromosome on which many essential immune related genes are located [49]. The additional X chromosome encodes genes involving regulatory T cells (Treg) function and development, which have roles in the pathogenesis of PAH [50,51,52,53]. In fact, a study of sex differences in Treg function found that, without the protection of Tregs, athymic female rats develop a more serious form of pulmonary hypertension induced by SU5416 and chronic hypoxia than male rats [54]. It has been also found that Treg dysfunction and dysregulation contribute to the growth and the progression of PAH [53]. Female rats lacking Treg cells had increased pulmonary inflammation, augmented RV fibrosis, and decreased vasodilation factors and vasoprotective proteins, resulting in increased endothelial/smooth muscle remodeling at the lung tissue [54]. These studies propose that females are significantly dependent on the regular role of Treg for compromising pulmonary vascular injury in pulmonary hypertension and that different strategies for PAH therapies such as Treg function stimulation might be applied for better outcomes for female PAH patients [54]. 

In addition, sex hormones including estrogen directly regulate mitochondrial function in different tissues involved in PAH pathogenesis. Estrogen and estrogen receptors α and β located in the mitochondria have been indicated in inflammation and apoptosis in PAH by promoting the transcription of nuclear respiratory factor-1 (*NRF-1*) to enhance mitochondrial transcriptional factors (*TFAM*) [55]. Farha and colleagues implied that mtDNA polymorphisms have a role in developing PAH for the first time [56]. The analysis of a Caucasian population (*n* = 204) and African American population (*n* = 46) found that mtDNA haplogroup M and its sibling haplogroup *n* that migrate out of Africa impose a greater risk of progressing PAH in comparison to the familial African haplogroup L. This means that progressing of the PAH disease is increased as an evolutional acquirement [56].

Furthermore, mitochondria and mitochondrial metabolism might have a critical role in the sex paradox in PAH. Mitochondria play a key role in the pathological mechanism of PAH by regulating vascular function and involving pulmonary vasoconstriction [49,57]. Indeed, excessive proliferative and antiapoptotic features in PAH are partly driven by vascular cell mitochondria [57]. Mitochondria can modulate the mitochondrial-derived reactive oxygen species which might diffuse to the cytoplasm and the plasma membrane to stimulate redox-sensitive targets such as Hif-1α or the NFAT signaling pathway causing pulmonary arterial smooth muscle cell contraction [57]. Mitochondria can also trigger the NLPR3 inflammasome, activating its downstream pathway that triggers the release of cytokines in PAH patients [58]. Importantly, T-cell activation and function are crucially linked to mitochondrial metabolism and function [59]. Interestingly, mitochondrial DNA, as well as stress-responsive mitochondria, can be passed on to the offspring [49]. These findings propose that female mitochondria are responsive to environmental and evolutionary changes which are subsequently passed on to the next generation, and that this notion can be explained by the sex paradox in PAH. 

Interestingly, studies have shown that DNA methylation regulates gene expression involving PAH in a female-biased manner noticed in PAH [60]. DNA methylation modification has a major role in deactivation of the X chromosome in females, and X chromosome loss might be involved in PAH pathogenesis [60,61]. Altogether, DNA methylation is significantly associated with the phenotype of monoallelic transcription; therefore, alterations in the methylated DNA pattern on the X chromosome might play a role in the “sex paradox” in PAH or anticipate sex-specific response to treatment agents such as DNMT inhibitors [61]. 

Taken together, PAH is a progressive and devastating cardiovascular disease that affects the female population more than the male population. Paradoxically, the mortality of PAH patients is greater in males. Many studies have been conducted to find out underlying mechanisms of sex discrepancy in PAH. However, further studies applying an interactive preclinical model are in need to demystify the involvement of sex hormones and their metabolites, as well as other epigenetic and signaling pathways in sex differences in PAH. 

Microchip systems with micron-sized channels that permit cells to grow and interact with each other in a three-dimensional structure imitating the biological environment of tissues in the body have been used to bridge the gap between in vivo and in vitro systems [62]. In a recent publication, Al-Hilal et al. from the Ahsan group used a microfluidic chip model simulating a PAH-insulted artery to study the sex discrepancy in PAH pathophysiology [63]. Chips composed of cells from male patients demonstrated decreased intimal (endothelial cells) but greater medial (smooth muscle cells) thickening compared to chips composed of cells from female patients [63]. It is implicated that male patients develop a more severe disease compared to female patients illustrated by chips with male cells, exhibiting a higher number of PASMCs with a FSP-1^+^CD31^−^ signature in the intimal and luminal layers [63]. β-Estradiol triggered a greater elevation in the thickness of intimal and medial layers in the chips with female cells when compared to the chips with male cells. In the chips with female cells, aromatase and CYP1B1 expression was greater than in those with male cells, despite the collagen deposition being greater in the latter [63]. This study suggests that the PAH chip design reacts to sex hormones in manner similar to that observed in PAH patients, and that it is a useful model for studying mechanism of sex disparity to advance sex-specific treatment for PAH patients.

## 4. Epigenetic Mechanisms of Pathogenesis in PAH

**Figure 3:**
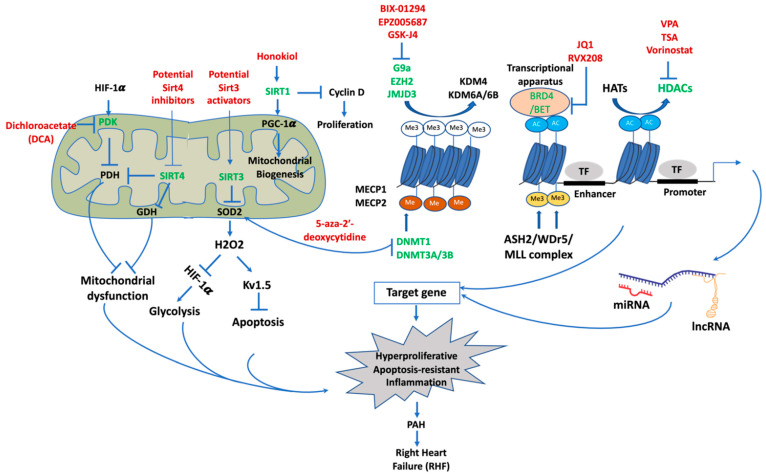
Potential epigenetic therapeutic targets for PAH. Targeting epigenetic modifications that involve PAH pathogenesis and right-heart failure to develop drugs for the treatment of PAH. Epigenetic mechanisms including demethylation of typical methylation motifs histone 3 lysine 27 trimethylation (H3K27me3) and histone 3 lysine 9 trimethylation (H3K9me3), DNA methylation, methylation of histone 3 lysine 4 (H3K4), and acetylation and deacetylation of histone lysine residues, as well as miRNAs and lncRNAs, in PAH affect chromatin packaging, accessibility, and gene expression and permit transcriptional elements and transcription apparatus to modulate gene transcription. Activation of enzymes, such as methyltransferases, demethylases, acetyltransferases, and deacetylases, is required for transitional states of chromatin for regulating gene expression. Sirtuins regulate apoptosis, glycolysis shift, and mitochondrial biogenesis in PAH. These processes lead to hyperproliferation, apoptosis resistance, and inflammation of PASMCs and PAECs, resulting in PAH pathology and ultimately right-heart failure. Epigenetic modifications that have been targeted for drug treatment of PAH are shown in green. Epigenetic modulators that were experimentally tested and potential modulators are listed in red. Me3, trimethylation; Me. methylation; Ac, acetylation.

Epigenetics refers to phenotype changes that can be heritable by alteration in the expression of genes, but not in DNA coding sequence [64]. These heritable alterations are specific epigenetic changes named epigenetic marks that affect chromatin packaging, accessibility, and gene expression [65]. Epigenetic modifications include DNA methylation, histone mechanisms, and noncoding RNAs (ncRNAs) (Figure 3). Dynamic homeostasis of histone remodeling, directed by the actions of modifiers and incorporation of downstream effectors, governs chromatin structure and is a major regulator of transcriptional responses in various cell types [66]. Three essential mediators of histone modifications are writers, erasers, and readers, which are the protein apparatus that add, remove, and recognize these changes [66]. Writers are enzymes that add specific modifications to histones. Similarly, enzymes that take away specific modifications from histone substrates called erasers. Lastly, readers are protein effectors that recognize either specific epigenetic traces on histones or a combination of traces and histone proteins to drive a particular gene expression [66]. Unlike mRNA, ncRNAs are not encoded for proteins or peptides, and they include siRNAs, microRNAs (miRNAs), long noncoding RNAs (lncRNA), circular RNAs (circRNAs), and extracellular RNAs [67]. ncRNAs modulate the expression of genes at the transcription and post-transcription levels [68]. The most extensively studied ncRNAs are miRNAs and small noncoding RNAs 22 nucleotides in length that monitor gene expression by degrading their target mRNAs and/or suppressing their translation [69]. lncRNAs are transcripts of nucleotides that are not translated into proteins comprising a heterogeneous class of intergenic, intronic, divergent, and antisense variations depending on their genomic location [70]. There is mounting proof showing that dysfunction of these epigenetic mechanisms has critical roles in human diseases including cancer, pulmonary arterial hypertension, and metabolic and cardiovascular diseases [71,72,73,74,75]. As such, hypomethylation is commonly observed in malignant cells related to chromosomal instability and loss of normal allele-specific gene expression [74]. Targeting epigenetic regulators including DNA methylation and histone post-translational modifications such as histone methylation (writer)/demethylation (eraser) and histone acetylation (writer)/deacetylation (eraser), as well as bromodomains (as histone acetylation readers), sirtuins (as major deacetylases), or miRNAs, offers many possible candidates for drug discovery and will no doubt be a tempting area for scientists and the pharmaceutical industry, especially for drug development in PAH therapy [74,76,77,78,79].

### 4.1. DNA Methylation

DNA methylation is a procedure of adding methyl groups (–CH_3_) to the DNA molecules at CpG or CG sites in the 5′ to 3′ direction, resulting in the formation of 5-methylcytosine [80]. DNA methylation functions in long-term transcriptional repression and in the formation of heterochromatin [81]. Methylation is driven by a family of DNA methyltransferases (DNMTs) including DNMT1, DNMT2, DNMT3A, DNMT3B, and DNMT3L that use *S*-adenosyl-methionine as the methyl donor [82]. DNA methylation modification allows these suppressed states to be transferred through cellular divisions [81]. 

Promoter regions of active genes are less efficiently methylated because of their modification in histone 3 lysine 4 trimethylation (H3K4me3) as the K4me3 tail attached to the enzymatic ADD domain preventing promoter methylation. However, cytosine-enriched areas have higher methylation percentages [83]. Binding of ADD to trimethylated H3K4 initiates the DNA methylation process by stimulating the MTase domain.

DNA methylation at CpG loci suppresses and turns off gene transcription [81]. Methylated cytosines act as docking sites for methylated CpG-binding proteins called MeCP1 and MeCP2 that particularly recognize and bind to methylated CpGs and suppress transcription indirectly via recruitment of corepressors that modify chromatin [84]. Alternately, MeCP2 or MeCP1 might create a complex together with other molecules to compress the chromatin [84]. Mutations in the MeCP2 gene lead to abnormal development of neurological system mostly in girls as Rett syndrome with mental retardation [85]. Another mechanism occurs where DNA methylation silences gene expressions by structural modifications directly incorporated with the methyl group, which obstructs the interconnection of the transcriptional factor on its DNA-binding positions, thus preventing the transcription of the gene [86]. In fact, CpG methylation promotes more compact wrapping of DNA around the histone core, associated with topology alteration [87]. In addition, methylated DNA coordinates with methyl-CpG-binding domain proteins (MBDs) to interact with other proteins including HDAC and additional chromatin-modifying proteins compressing and inactivating chromatin [88]. The role of CpG island hypermethylation of tumor suppressor genes has emerged in cancer development and progression [88]. 

In pulmonary arterial hypertension, DNA methylation has a key function in modulating the expression of superoxide dismutase 2 (SOD2), a major antioxidant protein enzyme localized in mitochondria. SOD2 level of expression is remarkedly reduced in PASMCs in individuals with PAH and induced PAH in Fawn-hooded rats (FHR) [89,90]. Indeed, suppression of SOD2 stimulates hypoxia inducible factor (HIF-1α) causing an enhancement in the expression level of voltage-gated K-channels (Kv1.5)—an oxygen-sensitive gene. These alterations cause impaired oxygen sensing and decrease redox status in cytoplasm and mitochondria [89]. Suppression of SOD2 transcription and translation using siRNA in normal PASMCs from Sprague–Dawley rats displays the PAH phenotypic features. In contrast, SOD2 induction decreases HIF-1α expression and re-establishes the expression of Kv1.5 in FHR PASMCs. In fact, genomic bisulfide sequencing has identified a selective hypermethylated CpG island in the promoter area and enhancer area of intron 2 of the SOD2 gene [90]. In particular, elevated DNA methylation has been found to be associated with enhanced expression level of DNMT1 and DNMT3B in PASMCs derived from PAH subjects and FHR rats [90]. Mostly, suppression of DNA methylation by 5-aza-2′-deoxycytidine (a DNA methyltransferase inhibitor) restores SOD2 level, decreases proliferation, and enhances programmed cell death in PASMCs derived from PAH induced by FHR [90]. These results suggest that epigenetic downregulation of mitochondrial SOD2 is attributed to the development of pulmonary arterial hypertension.

Heritable PAH (HPAH) represents 25–30% of all PAH cases [91]. Genetic studies have identified the presence of causal mutations of genes related to PAH development such as bone morphogenetic protein receptor (*BMPR2*), T-box 4 (*TBX4*), activin A receptor-like type 1 (*ACVRL1*), endoglin (*ENG*), small mothers against decapentaplegic homolog 9 (*SMAD9*), caveolin-1 (*CAV1*), potassium channel subfamily K member 3 (*KCNK3*), ATPase 13A3 (*ATP13A3*), SRY-box 17 (*SOX17*), aquaporin 1 (*AQP1*), growth differentiation factor 2/BMP9 (*GDF2*), and eukaryotic translation initiation factor 2 alpha kinase 4 (*EIF2AK4*) [91]. *BMPR2* genetic mutations are the most important risk factors for PAH, with a female penetrance of 42% and male penetrance of 14% [92]. It belongs to the TGF-β family and the BMP/TGF signal mechanism that stimulates bone formation and cellular differentiation [93]. Interestingly, Liu et al. showed that the BMPR2 promoter is hypermethylated, causing reduced *BMPR2* expression, in HPAH patients [94]. In addition, a low level of *BMPR2* expression in endothelial cells in lung tissue enhances cell proliferation and stimulates PAH development [94]. In contrast, Poussada et al. found no proof of methylation in PAH patients and controls by investigating the methylation pattern of the BMPR2 promoter area of genomic DNA isolated from the peripheral blood using methylation-specific PCR [95]. This discrepancy might be because only a few patients from diverse PAH groups were enrolled in these studies and different areas of the BMPR2 promoter or gene were analyzed [94,95]. Further studies are needed for identifying the methylation pattern of the *BMPR2* gene. As aforementioned, DNA methylation, as a critical modification of X chromosome deactivation in females, likely impacts PAH gene expression in females, possibly explaining the sex difference in PAH [60,61] 

### 4.2. Histone Post-Translational Modifications

Histones have a high degree of conservation and are the main constituents of chromatin structure [96]. The histone octamer is consisted of core histone dimers including H2A, H2B, H3, and H4 [97,98]. Genomic DNA is enclosed around the histone octamer and creates the nucleosome, which is identified as the chromatin repeating unit [98]. Histone post-translational modifications govern gene patterns by stimulating or suppressing gene expression. The most thoroughly studied histone modifications are histone acetylation and methylation [99]. There are also many lesser-studied histone modifications including glycosylation, biotinylation, SUMOylation, phosphorylation, ADP-ribosylation, and ubiquitination, which might also influence chromatin condensing [99]. More recently, emerging studies have identified other modifications on histones known as “acylations” by mass spectrometry, including propionylation, butyrylation, crotonylation, succinylation, malonylation, and 2-hydroxyisobutyrylation [100]. These newly discovered modifying mechanisms add a diverse spectrum of biochemical surroundings to histones modifying gene promoters or transcriptional regions that regulate gene activity, similar to acetylation. However, the physiological relevance and the regulation of these newly defined acylations on histone remain to be further determined [100]. Histone methylation and acetylation result in the incorporation of acetyl or methyl groups to lysine and/or arginine residues located on the histones [100,101]. 

#### 4.2.1. Histone Methylation and Demethylation and Targeting Histone Demethylase for Treatment of PAH

Histone methylation is a dynamic and reversible process. Histone methyltransferases (HMTs) and histone demethylases (HDMs) have been demonstrated to modulate the adding and removing of methyl groups from various lysine residues on histones, respectively [101]. Two evolutionarily preserved families of histone demethylases are the lysine-specific demethylases (LSD) and the Jumonji C family (JmjC), which utilize various reaction mechanisms for demethylation [102]. The lysine-specific demethylases (LSD) use FAD as a cofactor for its demethylation activity, while the Jumonji C family (JmjC) requires Fe^2+^ and α-ketoglutarate (α-KG) for its function [102,103]. 

One, two, or three methyl groups can be added to the ε amine group on lysine residues, while arginines can be monomethylated (me1), symmetrically dimethylated (me2s), or asymemetrically demethylated (me2a) on their guanidinyl group [101]. The most intensively defined histone methylation patterns are histone H3 lysine 4 (H3K4), H3K9, H3K27, H3K36, H3K79, and H4K20, while positions of arginine methylation are H3R2, H3R8, H3R17, H3R26, and H4R3 [101]. However, the histone proteins H1, H2A, H2B, H3, and H4 have also been methylated on many other basic residues as recently determined by mass spectrometry and quantitative proteomic analyses. The physiological relevance and the regulation of the recent discovered methylation occurrences remain to be identified [101].

Histone methylation has been indicated in the pathophysiological mechanisms of PAH [104,105]. Induced gene expression of cell adhesion molecules (CAM) has a key role in the pathology of PAH induced by chronic hypoxia. Activation of CAMs promotes leukocyte adherence to the vascular endothelium, stimulating inflammation [105]. Cell adhesion molecules (CAMs), including intercellular adhesion molecules (ICAMs), vascular cell adhesion molecules (VCAMs), and selectins, are a group of transmembrane proteins that mediate cell–cell interactions [105]. It has been identified that MRTF-A communicates with NF-κB, recruiting H3K4 methylase to the CAM promoters to induce CAM transactivation in response to hypoxic stress during hypoxia-induced PAH. Transcriptional upregulation of CAM gene expression leads to the activation of H3K4me3 as a marker of transcription activation resulting in PAH [105]. Furthermore, endothelial-specific downregulation of ASH2 and WDR5, two major constituents of the H3K4 methyltransferase, blunted recruitment of H3K4me3 at CAM promoters, causing a remarkedly decreased CAM expressional level improving hypoxia-induced PH in mice [105]. 

While H3K4me3 causes transcriptional activation, trimethylation of H3K9results in transcriptional suppression. G9a, an important enzyme for dimethylation of histone H3K9, has been found to be involved in ovine fetal PASMC proliferation, migration, and contractibility [106]. Accordingly, inhibition of G9a by its inhibitor BIX-01294 decreased the migration, proliferation, and contractility of fetal PASMCs in relation to a significantly increased global methylation level in the fetal PASMCs [106]. The expression level of the enhancer of zeste homolog 2 (EZH2) is increased in PASMCs isolated from PAH animals, leading to enhanced hypertrophy, right-ventricle systolic pressure, and PAH development [107]. Similarly, the upregulation of EZH2 in human PASMCs increases migration and proliferation, as well as reduces apoptosis [107]. Shi et al. identified that pharmacologic suppression of EZH2 with EPZ005687 improves transverse aortic constriction (TAC)-induced PAH by blocking ROS generation in the pulmonary system [108]. In fact, EPZ005687 remarkably reduced the H3K27me3 enhancement in the promoter area of SOD1, suppressing its transcriptional expression.

In another study, the role of histone H3 trimethylation on lysine 27 (H3K27me3) was investigated in the proliferation of PAECs, apoptosis, and inflammation in PAH [109]. It was demonstrated that pharmacologic suppression of JmjC domain-containing protein JMJD3 by GSK-J4 markedly reduced proliferation, enhanced programmed cell death, and decreased inflammatory mediator IL-6 in PAECs [109]. Additionally, JMJD3 demethylates H3K27me3, suggesting that JMJD3 might modulate the activation of pulmonary vascular endothelial cells via modulating H3K27me3 in PAH [109]. 

Histone modifiers proved to be appealing treatment strategies considering their capability to suppress or stimulate genes. Blockers of lysine-specific histone demethylase 1A (LSD1), an H3K4/K9 demethylase, are presently under evaluation in clinical trials for treatment of diseases [110]. Deficiency of LSD1 in heterozygous LSD1 knockout mice is related to hypertension, increased vascular constriction, and decreased relaxation through the NO-cGMP signaling pathway [111]. Interestingly, NO-cGMP signaling has been implied in modulating pulmonary vascular tone and is targeted by current PAH pharmacologic agents [112]. 

#### 4.2.2. Histone Acetylation and Deacetylation

Histone acetylation happens on lysine (K) residues at the amino ends of the four core histones and is regulated by the histone acetyltransferases (HATs) [113]. Histone lysine acetylation is an important epigenetic modification for regulating the transcriptional level of genes. Acetylated histones are less tight and more available to enzyme RNA polymerase for transcription, thus promoting gene expression [114]. Furthermore, acetylated histones provide binding loci for BRDPs to acquire transcription apparatus and additional chromatin remodeling factors [113,115]. 

In contrast to histone acetyltransferases HATs, HDACs detach acetyl groups from histone proteins and suppress the DNA transcription process by compacting the chromatin [116]. There are 18 human HDACs categorized into four classes in agreement with criteria of phylogenesis and function [116]. They are class I Rpd3-like proteins (HDAC1, HDAC2, HDAC3, and HDAC8), class II Hda1-like proteins (HDAC4, HDAC5, HDAC6, HDAC7, HDAC9, and HDAC10), class III Sir2-like proteins (SIRT1, SIRT2, SIRT3, SIRT4, SIRT5, SIRT6, and SIRT7), and class IV proteins (HDAC11) [116]. HDACs have substrate specificity similar to HATs and can also deacetylate nonhistone proteins [116]. HDAC enzymes are different in terms of configuration, enzymatic activity, subcellular colocalization, and expression. In addition to deacetylating histone, HDAC isoforms can regulate numerous cytoplasmic proteins, including transcription factors, via controlling their acetylation level [117]. 

Bromodomains (BRDs) are conserved structures that read and attach to lysine residues, being acetylated on histone and non-histone proteins, interpreting the histone acetylation topography [118]. They mainly serve as epigenetic “readers” in coordination with protein “writer” HATs and protein “eraser” HDACs to mediate histone acetylation marks and regulate gene expression via multiple mechanisms [118]. BRDPs are clustered phylogenetically into families including (a) direct chromatin modifiers containing an intrinsic lysine acetyltransferase in addition to their BRD domain such as EP300/CREBBP or lysine methyltransferase such as KMT2A/ASH1L, (b) chromatin remodelers containing a bromodomain and an ATPase domain such as SMARCA2/SMARCA4, and (c) “bromodomain and extra-terminal domain” (BET) with two BRDs and an extra-terminal domain [118,119]. Among BET members BRD2, BRD3, BRD4, and the testis-specific isoform BRDT, BRD4 has been found to assist with telomere elongation and DNA repair by upregulating DNA repair factors [120,121].

##### Targeting Histone Deacetylase for PAH Therapy

HDAC inhibitors, including class I HDAC inhibitor, valproic acid, and class I, II, and IV HDAC inhibitor suberoylanilide hydroxamic acid (vorinostat), have been found to alleviate the growth of established hypoxia-induced PAH in rats and reduce the proliferation of human PASMCs and human PAECs, as well as inflammation in fibroblasts and R-cells [122]. Moreover, this study also found that the protein levels of HDAC1 and HDAC5 are remarkably overexpressed in the pulmonary system from human IPAH and in the pulmonary system and right-ventricular heart from hypoxia-induced PH rats [122]. In line with this finding, another study found that epigenetic histone acetylation regulates the levels of *eNOS* expression, which has a critical role in the pathogenesis of persistent PH of the newborn (PPHN) [122]. Accordingly, the acetylation extent of H3 and H4 in the promoter region of the *eNOS* gene is elevated in pulmonary vascular endothelial cells from PPHN [122]. More recent publications have shown the important function of HDAC inhibitors in RV failure in in vitro and animal models of PH. Effects of sodium valproate, an HDAC blocker, on induced-RVH in rats by PAB or MCT injection were assessed by Cho et al. [123]. MCT is well known to selectively induce pulmonary endothelial injury, which causes RVH secondary to pulmonary arterial hypertension [123]. It has also been identified that oral administration of sodium valproate caused an increase in histone acetylation, leading to effective blockage of RVH induced by PAB or MCT injection, suggesting that HDAC inhibitors might be a potential new therapeutic strategy for RVH secondary to PAH [123]. Additionally, Lan et al. identified that valproic acid (VPA) treatment could impede and partly inverse severe PH using MCT and a chronic hypoxia combination method [124]. VPA inhibits HDAC1 resulting in histone 3 hyperacetylation, thereby regulating proliferation, inflammation, and programmed cell death in modified pulmonary vasculature [124]. In another study, Bogaard et al. investigated the effects of trichostatin A (TSA) and valproic acid (VPA) as wide-spectrum HDAC inhibitors on RV role and modification after PAB in rats [125]. Controversially, this group showed that inhibition of HDAC with TSA failed to block the development of RV remodeling, but was rather associated with RV fibrosis, RV dysfunction, and increased myocardial cell death [125]. The discrepancy between Cho et al. and Bogaard et al. in the results of valproic acid on PH experimental animal models was explained by the two groups applying different strategies in the use of animal (young rats by Cho et al.) and in the initiated treatment (directly after surgery by Cho et al. versus 4 weeks after surgery by Bogaard et al.) [125].

HDAC inhibitors such as scriptaid, suberoylanilide hydroxamic acid (SAHA), trichostatin A (TSA), and valproic acid (VPA) have been revealed to decrease Nox expression and ROS production via reducing histone activation traces (H3K4me3 and H3K9ac) in the Nox 2, 4, and 5 promoter regions, thus mitigating indices of PAH in the MCT rat model [126]. Indeed, they prevent the attachment of RNA polymerase II and HAT p300 to the Nox2, Nox4, and Nox5 promoter regions [126]. 

In addition, pharmacological inhibition of class IIa HDACs by their selective inhibitor MC1568 has been reported to restore MEF2 function in PAECs by enhancing its target transcription, reducing cell migration and proliferation, and reversing characteristics of experimental PAH [127]. The function of the transcriptional factor myocyte enhancer factor 2 (MEF2) was identified to be remarkedly blunted in the PAECs obtained from PAH patients [127].

Cavasin et al. tested two selective inhibitors of class I HDACs (HDAC1, HDAC2, and HDAC3), MGCD0103 and MS-275, in the hypoxia-induced PH rat model. Both compounds reduced pulmonary arterial pressure animals with diminished thickening of the pulmonary arterial wall via repressing the proliferation of smooth muscle cells [128]. They moderately reduced right-ventricular hypertrophy, suppressed pathological gene expression and proinflammatory protein expression, and repressed proapoptotic caspase activity [128]. Yang et al. demonstrated that HDAC inhibition with apicidin reduces RVH and pulmonary vascular modification via reducing the chronic hypoxia-induced activation of the IGF-1/pAKT signaling pathway in the lung tissue of neonatal mice [129]. Thus, HDAC inhibitors might be promising for pediatric and adult onset of PAH via numerous molecular signaling pathways.

More recently, Boucherat et al. found that HDAC6 is involved in the pathogenesis of PAH. Its expression level is significantly increased in pulmonary tissue, distal pulmonary arteries, and PASMCs derived from PAH individuals and PAH induced by MCT and sugen/hypoxia in animals [130]. In addition, pharmacological inhibition of HDAC6 by Tubastatin A (TubA) or ACY-775 or siHDAC6 remarkably reduced the proliferation and migration of PAH PASMCs and improved apoptosis resistance. Blockage of HDAC6 also ameliorated PAH induced in the sugen/hypoxia and MCT rat model as illustrated by reducing abnormal high right-ventricle systolic and pulmonary pressure, as well as right-ventricle and vascular remodeling [130]. Furthermore, Hdac6 knockout mice were partly protected from pulmonary hypertension induced by chronic hypoxia [130]. These findings demonstrated that HDAC6 is indicated in PAH pathogenesis and is a potential target for PAH therapy.

In short, HDAC has a key role in the pathogenesis of PAH, and HDAC inhibitors have been demonstrated as potential pharmacologic agents to improve vascular and RV remodeling, including reduced proliferation, induced apoptosis, and suppressed inflammation in induced PAH using experimental animal or cell culture models. However, as aforementioned, there is a disagreement on the effects of HDAC inhibitors in the treatment of induced PAH. First, this might be because of the variability and severe level of RV remodeling among studies, which can be in different stages of adaptation and compensation in response to disease state. It is likely that the treatment of HDAC inhibitors could improve or worsen the RV remodeling depending on the stages of the remodeling process, as similarly described using HDAC inhibitors for the treatment of patients with lymphoma based on severity [131]. Second, selective inhibitors of HDACs yield better outcomes on selective targets and fewer side-effects on off-targets compared to nonselective HDAC inhibitors [127,128]. Third, the usage of various PAH models (sugen/hypoxia, MCT, and PAB), as well as initiation, duration, and frequency of treatment, may affect the results of HDAC inhibitors in experimental models. Regardless of these factors, HDAC inhibitors are a promising therapeutic approach for PAH. It is likely important to classify stages or types of RV remodeling in PAH for an efficient corresponding treatment. 

##### Targeting Bromodomains as Histone Acetylation Readers for PAH Therapy

BRD4 is overexpressed in pulmonary tissue, distal lung arteries, and PASMCs derived from PAH subjects compared to control [132]. Moreover, inhibition of BRD4 by JQ1 or siBRD4 causes a reduced expression of nuclear factor of activated T cells (*NFAT*), B-cell lymphoma 2 (*BCL2*), and *survivin*, which are three important oncogenes [132]. Inhibiting this tumorigenic gene signature results in reduced PAH PASMC proliferation and enhanced apoptosis in a BRD4-dependent fashion. In addition, experimental inhibition of BRD4 inversed PAH indices in the sugen/hypoxia rat model [132]. Mechanistically, this study also found that microRNA-204 (miR-204) increased BRD4 expression in PAH [132]. In another study, FOXM1 caused an increase in PAH PASMC proliferation and apoptosis resistance regulated by miR-204 expression [119]. Pharmacological inhibition of FOXM1 ameliorates PAH induced by MCT and chronic hypoxia in the MCT and Su/Hx rat models [119]. In a recent study, Van der Feen reported that BRD4 is overexpressed in the modified pulmonary vessels of PAH individuals through interactions with FoxM1 and PLK1, which are involved in the responsive mechanism to DNA damage. Inhibition of BET by BET inhibitor RVX208 restored the abnormal phenotypes of proliferation, apoptosis resistance, and inflammation of PAECs and PASMCs derived from PAH subjects [133]. Furthermore, RVX208 inversed vascular modifying and ameliorated lung hemodynamics in the sugen/hypoxia and monocrotaline (MCT) + shunt-PAH rat models [133]. Additionally, RVX208 treatment reduced the pressure load to the right ventricle in the PAH induced by PAB rat model [133]. These findings suggest that BRD4 and FOXM1 could be novel therapeutic targets in PAH.

### 4.3. Sirtuins and Targeting Sirtuins for PAH Therapy

Sirtuins are class III HDACs that de-acylate protein substrates from lysine residues to regulate their activities, using nicotinamide adenine dinucleotide (NAD^+^) as a cofactor [134,135]. They are located in different cellular compartments with different levels of expression, including nucleus sirtuins (Sirt1, Sirt6, and Sirt7), cytosol sirtuins (Sirt2), and mitochondrial sirtuins (Sir3, Sirt4, and Sirt5), in addition to being diverse in terms of substrate specificity and post-translational modifications of histone and non-histone targets [136]. Sirtuin members have NAD^+^-dependent de-acylase, and ADP-ribosyltransferase enzymatic functions, which are responsible for cellular activities such as proliferation, differentiation, apoptosis, senescence, mitochondrial biogenesis, metabolism, aging, and inflammation [137]. Sirt1 is the most thoroughly studied among nucleus sirtuins [138] and Sirt3 is the most extensively studied member among mitochondrial sirtuins [139]. The role of sirtuins in PAH, their expression levels, and the lysine acetylation level in human PAH and experimental models have not yet been revealed in detail. Several publications have shown that sirtuins might exert a protective capacity by improving PH and RVH, especially Sirt1 and Sirt3 in animal models.

Recently, Zurlo et al. demonstrated that Sirt1 inactivation altered the acetylation/deacetylation balance implied in the pathologic process of PAH [140]. Accordingly, inhibition of Sirt1 by pharmacologic agent or short-interfering RNA potentiated proliferation of PASMCs isolated from rat and human. In addition, Sirt1 knockout mice displayed vascular remodeling, accompanied by an elevation in RV pressure and hypertrophy [140]. Interestingly, Sirt1 activation by Stac-3 reduced acetylated histone H1 and Forkhead box protein O1 (FOXO1) and strikingly reduced the proliferation of PASMCs derived from rat and human without influencing cell death because of increased mitochondrial biogenesis [140]. In another study, Ding et al. found that CR improved MCT-induced mPAP and decreased pulmonary arterial remodeling and right-ventricle hypertrophy. These useful effects of CR were found to be related to the normalized SIRT1 expression level and phosphorylation state of endothelial nitric oxide synthase (eNOS) and the decreased eNOS acetylation level in pulmonary arteries of PAH-induced rats [141]. Significantly, Sirt1 overexpression using adenoviral vectors in MCT-induced PAH and hypoxia-induced PH in rats showed similar beneficial effects on the reduction in mPAP and eNOS acetylation as obtained merely with short-term CR [141]. These findings demonstrated that the protective role of Sirt1 during CR prevented vascular remodeling and hypertrophy of the right-ventricle heart in PAH experimental models, and that Sirt1 activators could be promising pharmacologic agents for PAH treatment.

Growing evidence suggests that mitochondrial dysfunction involving alterations in metabolic state, apoptosis, and mitophagy plays pivotal roles in the pathogenic process of PAH [142]. Sirt3 is a major NAD^+^-dependent deacetylate enzyme located in the mitochondria. Sirt3KO mice displayed phenotypes of cardiac hypertrophy and interstitial fibrosis compared with wild-type controls [143]. Sirt3 inhibited cardiac hypertrophy by stimulating the Forkhead box O3a-dependent (Foxo3a-dependent), manganese superoxide dismutase (MnSOD) or superoxide dismutase 2 (SOD2) and catalase (Cat), thus suppressing cellular levels of ROS in primary cardiomyocytes [143]. These findings indicate that Sirt3 negatively regulates cellular ROS levels to suppress cardiac hypertrophy [143]. It has been established that downregulation of mitochondrial function increases proliferation and apoptosis resistance in the pulmonary arteries and other tissues in PAH; however, the etiology of this metabolic dysregulation is unknown. Paulin et al. reported that mice lacking Sirt3 have reduced mitochondrial function by enhancing acetylation level, which leads to decreased activity of multiple enzymes and protein complexes in the mitochondria [144]. Furthermore, Sirt3 is significantly downregulated in tissues derived from PAH-induced by MCT and PAH subjects, while restoring Sirt3 using adenovirus reverses the PAH phenotypes [144]. Sirt3KO mice develop spontaneous PAH and display enhanced muscularization and thickening of the medial wall of insulted pulmonary arteries in comparison to the littermate controls [144]. 

However, a study by Waypa et al. yielded different results of Sirt3 deletion on PA remodeling and RVH, when challenged to chronic hypoxia [145]. Sirt3 deficiency mice did not change HIF-1α stabilization under normal conditions and in chronic hypoxia compared to littermate controls. Moreover, Sirt3^(−/−)^ mice developed PH with remodeling of pulmonary arteries and hypertrophy of the right-ventricle heart similarly to wildtype littermate control mice. The discrepancy in results between the two studies might come from the cell type and the specific genetic background of mice in response to Sirt3 [145].

Interestingly, treatment with pyruvate dehydrogenase kinase (PDK) inhibitor dichloroacetate (DCA) can improve mitochondrial respiration ex vivo via activating PDH and reducing mean PA pressure and pulmonary vascular resistance in iPAH patients already on approved therapies, albeit with variations in individual responses [146]. The reason for not detecting ex vivo and clinical response was attributed to polymorphism in Sirt3 and UCP2 [146]. These results suggest that response to PAH treatment varies depending on multiple factors, and personalized therapy might offer a better outcome for PAH. 

A recent study proved that bone marrow-derived exosomes improve mitochondrial function in PAH [147]. In particular, this study found that mesenchymal stromal cell-derived exosome exposure enhanced pyruvate dehydrogenase complex (PDH) and glutamate dehydrogenase (GDH), reversing the mitochondrial dysfunction of PAH, which was associated with reduced SIRT4 expression [147]. Furthermore, this study also showed that prolonged hypoxia induced SIRT4 expression, an upstream inhibitor of both PDH and GDH [147]. In fact, SIRT4 has been previously indicated to downregulate glutamate dehydrogenase (GDH) [148] and pyruvate dehydrogenase complex (PDH) [149]. It is possible that SIRT4 has a major function in the pathogenesis of PAH, thus potentiating exosomes for PAH therapy.

Resveratrol (3,5,40-trihydroxy-trans-stilbene), a sirtuin activator found in red wine and grape skins as a natural phytoalexin, has been found to improve pulmonary arterial remodeling in chronic hypoxia-induced PAH and MCT-induced PAH [150,151,152,153]. Indeed, resveratrol abrogated hypoxia-induced human PASMC proliferation by suppressing arginase II and proliferation in human PASMCs mediated via the PI3K/Akt signaling pathway [150]. In addition, resveratrol treatment improved the systolic pressure of right-ventricular heart and remodeling of pulmonary arteries, reduced the expression level of inflammatory mediators, and inhibited PASMC proliferation [152]. Resveratrol-treated rats displayed enhanced activity of endothelial NO synthase, reduced oxidative stress, and ameliorated function of PAECs [152]. In another study, activation of Sirt1 by resveratrol attenuated PA remodeling and pressure, as well as p21 expression, via mediating the cell cycle and inhibiting the expression level of cyclin D1 in MCT-induced PAH rats [154]. Furthermore, resveratrol via mediating Sirt1 could improve the severity of PAH by reversing the remodeling of the pulmonary vasculature in hypoxia-induced PAH and inhibiting PASMC proliferation [155]. Sirt1 activation by its specific activator SRT1720 alleviates RVSP and RVH, results in significantly reduced PASMC proliferation, and promotes PASMC apoptosis [155]. 

In short, resveratrol provides some beneficial effects on the pulmonary arteries including anti-inflammation, antioxidant, and antiproliferation, which might be applied for preventing pulmonary arterial hypertension as a potential future therapy. Sirtuin activation (Table 1) might be a promising strategy to further explore new therapeutic agents for PAH.

#### 4.3.1. Noncoding Ribonucleic Acids (ncRNAs)

Non-translating RNA molecules are usually known as a noncoding RNAs (ncRNAs). Only 2% of the human genome is translated into protein, while the rest is ncRNA [160]. On the basis of their functional activities, ncRNAs are classified into two types: functional ncRNAs such as microRNAs (miRNAs), circular RNAs (circRNAs), and long noncoding RNAs (lncRNAs) and non-functional ncRNAs such as junk RNAs. ncRNAs that are functional regulate the cellular pathways and, therefore, enable modulating the initiation of diseases and their progression. A few decades ago, among several species of ncRNAs, the roles of miRNAs and lncRNAs were widely investigated in disease pathologies, including pulmonary arterial hypertension (PAH), cancer, and neurodegenerative diseases [161,162].

#### 4.3.2. Role of miRNAs in PAH

A miRNA is a short single-stranded endogenous RNA molecule (approximately 22 nucleotides long) that is highly found in most human cells. Half of all protein-coding messenger RNA (mRNA) is regulated by miRNAs. They are usually derived from primary miRNAs (pri-miRNAs); after maturation, they mainly stay in the cytoplasm, where each mature miRNA becomes involved in the RNA-induced silencing complex (RISC) and then interacts with its mRNA target. Upon binding to an mRNA, the miRNA RISC promotes the silencing of the mRNA-encoded protein via one or more of the following mechanisms: (a) splitting of the mRNA strand into two fragments; (b) making the mRNA unstable via truncating of its poly (A) tail; (c) ineffective translation of the mRNA into proteins by ribosomes [162]. 

The expression of miRNA is one of the major mechanisms for epigenetic regulation [163,164]. Since gene expression is governed by miRNAs via a decrease in the translation of target mRNAs, they play a major role in PAH, a disease that is characterized by excessive proliferation and resistance to apoptosis of PASMCs, PAECs, and pulmonary arterial adventitial cells (PAADCs). Mounting evidence has established the role of dysregulated miRNAs in the hyperproliferative and apoptosis-resistant phenotype of pulmonary vascular cells, including PAECs and PASMCs, in various PAH models [162]. Several major dysregulated miRNAs are involved in PAH pathogenesis, as shown in Table 2. 

#### 4.3.3. Role of lncRNAs in PAH

lncRNAs are a class of ncRNAs greater than 200 nucleotides in length, which are synthesized by RNA polymerase II. They are mainly located in the nucleus of the cell, along with a low level of expression. They play an important epigenetic role by functioning as suppressors or activators of gene transcription and mRNA translation, miRNA precursors, RNA stabilizers, and sponges [165]. Emerging evidence has demonstrated a link between lncRNAs and the initiation of various human diseases, including cancer and cardiovascular and neurodegenerative diseases [165]. Pathological changes of PAH include excessive cell proliferation, migration, and a loss of balance between cell growth and death induced by apoptosis. Substantial evidence has indicated that these pathological features are regulated by lncRNAs and their mechanisms of action. These PAH features and their differentiation are also regulated by lncRNAs. Most PAH features are similar to common features of cancer, such as excessive cell proliferation and apoptosis resistance. Thus, lncRNAs are associated with not only PAH but also different types of cancer [161]. Several important lncRNAs are identified in PAH, and their important functions for the development of PAH pathologies are shown in Table 3. Although a large number of lncRNAs have been identified, their application for PAH treatment remains elusive. Thus, accelerating translational research will help us to develop a new and successful therapeutic PAH modality in near future.

#### 4.3.4. Therapeutic Potential of miRNAs and lncRNAs 

##### miRNAs

The therapeutic potential of miRNAs is still at a preclinical stage, although miRNAs have exhibited a promising role in PAH therapies. There are mainly two avenues through which miRNA-based therapy works. One is to elevate the downregulated miRNAs by using miRNA mimics, and the other is to inhibit the upregulated miRNAs through the use of antagomirs, masking, sponges, and erasers [166]. However, there still remain several challenges to successfully incorporate these approaches into the clinic. One challenge is to prepare the exact animal models for testing the miRNAs (mimics and antagomirs) in a closely similar human PAH phenotype. Another challenge is that miRNAs are unstable and nonspecific. Like siRNAs, the stability of miRNAs is improved by encapsulating miRNAs within lipid-based nanoparticles. However, when the treatment is intravenously administered, miRNA-loaded nanoparticles accumulate into several tissues via the passive or active pathway. Through the passive strategy, miRNA-loaded nanoparticles mainly internalize into the cells of the liver, spleen, and lymph nodes, whereas the active strategy helps nanoparticles to internalize into the cells of interest. Thus, toxicities are reduced by using the active strategy; however, owing to the short seed sequence of miRNAs, there is no way to completely remove the off-target effects of miRNAs. 

**Table 2:** List of important dysregulated miRNAs in PAH.

Cell Types/Animal Models	miRNA	Target mRNA	Function of miRNA	Expression	References
Human and rat PASMCs	miR-1281	Phosphatidylinositol 3-kinase–DNA methyltransferase 1–miR-1281–histone deacetylase 4	Antiproliferation	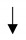	Y Li et al., J Am Heart Assoc. 2018 [167]
HPAH and IPAH BOECs Rat SUGEN-hypoxia model of severe PAH	miR-124	PTPB1 and PKM2	Proliferation	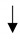	P Caruso et al., Circulation 2017 [168]
Hypoxic human PASMCs	miR-140	miR-140-5p–DNMT1–SOD2	Proliferation	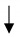	Y Zhang et al., Biochem Biophys Res Commun. 2016 [169]
Human PASMCs PAH patients and in preclinical models of PAH.	miR-34a	miR-34a-3p–MiD-DRP1	Proliferation and anti-apoptosis.	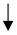	KH Chen et al., Circulation 2018 [170]
Human PASMCs Sugen/hypoxia rat model	miR-204	miR-204–BRD4	Proliferation	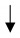	M Meloche et al., Circ Res. 2015 [132]
Endothelial cells (PAECs)	miR-424 and 503	FGF2 and FGFR1	Proliferation	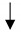	J Kim et al., Nat Med. 2013 [171]
Human PAH lungs, distal PAs, and isolated PASMCs	miR-223	PARP-1	PASMC proliferation	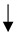	J Meloche et al., Am J Physiol Cell Physiol 2015 [172]
Human PAH PAECs	miR-17/92	BMPR2	PAEC survival	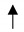	M Brock et al., Circ Res. 2009 [173]

Abbreviations: PAH, pulmonary arterial hypertension; IPAH, idiopathic pulmonary arterial hypertension; HPAH, heritable pulmonary arterial hypertension; BOECs, blood outgrowth endothelial cells; PASMCs, pulmonary artery smooth muscle cells; PAECs, pulmonary arterial endothelial cells; PTBP1, polypyrimidine tract-binding protein; PKM2, pyruvate kinase 2; DNMT1, DNA methyltransferase 1; SOD2, superoxide dismutase 1; MiD-DRP1, mitochondrial dynamic-related protein 1; BRD4, bromodomain-containing protein 4; FGF2, fibroblast growth factor 2; FGFR1, fibroblast growth factor receptor 1; PARP1, poly [ADP-ribose] polymerase 1; BMPR2, bone morphogenetic protein receptor type II.

**Figure 4:**
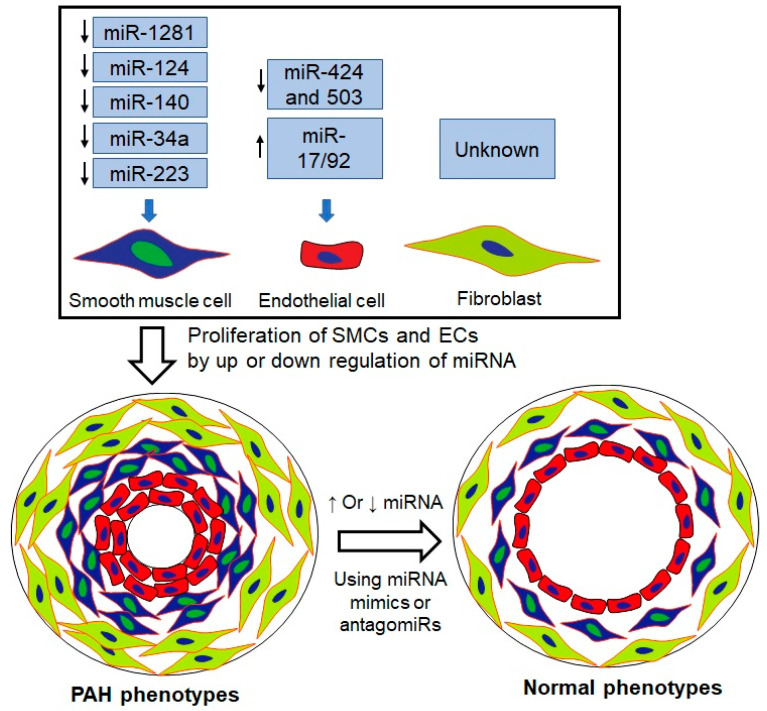
Targeting miRNAs can revert the phenotype of PAH to normal by inhibiting cell proliferation. During PAH pathogenesis, the expression of several miRNAs (miR-1281, miR-124, miR-140, miR34a, and miR-223) in smooth muscle cells of pulmonary artery is downregulated, while that of others (miR-424 and 503 and miR-17/92) is down and upregulated in endothelial cells. However, miRNA expression in the fibroblast still remains to be clarified. Under up- and downregulated conditions, the proliferation of cells, including smooth muscle, endothelial, and fibroblast cells within the pulmonary artery, occurs and, in turn, narrows the artery and decreases blood blow, while increasing the pressure, leading to PAH pathogenesis. Delivery of miRNA mimics to the downregulated miRNAs or that of antagomiRs to the upregulated miRNAs in these cells could be a possible way to return the situation to normal.

During PAH pathogenesis, several miRNAs are upregulated or downregulated. Thus, a single miRNA is not sufficient to prevent or regress PAH. Among various identified miRNAs responsible for PAH, there are four miRNAs, miR-29, miR-124, miR-140, and miR-204, that may be beneficial, because they have a conserved expression pattern in animal models and PAH human tissues [162]. A combination of convergent miRNA targeting may be an effective therapeutic approach compared to monotherapy. Although there are several challenges in PAH therapy, preclinical studies have shown a promising result for the clinical success of miRNA therapeutics in the future (Figure 4).

##### lncRNAs

Although many lncRNAs have been detected in PAH patient samples and preclinical animal models [165], there are no clinical trials targeting lncRNAs in PAH that have been developed. There are several reasons, including poor conservation across the species, the stability of lncRNAs in blood circulation, the lack of proper drug delivery systems, routes, and doses, and nonspecific toxicities. One of the major challenges for the clinical success of lncRNAs includes the species-to-species variation that hinders the development and testing of new drugs for clinical translation to human. As the expression of lncRNAs is highly variable for each cell/tissue, developing personalized treatments could be a way to achieve clinical success. A study demonstrated that combined treatment of BC-819 (a double-stranded DNA plasmid containing a gene for diphtheria toxin under the regulation of the H19 gene promoter) and *Bacillus* Calmette–Guerin showed potential to significantly ameliorate patient outcomes (e.g., 54.1% recurrence-free bladder cancer survival rate and 75.7% progression-free survival rate over 24 months in a phase II clinical trial in comparison to patients with non-muscle-invasive bladder cancer) [174]. As discussed above for the miRNAs, a proper delivery system also needs to be developed for lncRNAs that can improve their stability in blood circulation and minimize the nonspecific toxicities. 

**Table 3:** Summary of important lncRNAs involved in PAH pathogenesis.

lncRNAs	Cells/Animal Models	Targets	Functions	Expression	References
MEG3	Human PASMCs	miR-21/PTEN; p53 pathway	Inhibits proliferation and migration	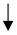	Zhu B et al., Biochem. Biophys. Res. Commun. 2018 Sun Z et al., Cell Physiol. Biochem. 2017 [175,176]
MEG3	Human PASMCs	miR-328- 3p/IGF1R	Proliferation of PASMC under hypoxia	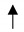	Xing Y et al., Mol. Ther. 2019 [177]
LNCRNA-ANG362	Human PASMCs	NF-κB-miR-221 and miR-222	Proliferation and migration of HPASMCs	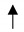	Wang H et al., SHOCK 2020 [178]
TYKRIL	Human PASMCs	p53/PDGFR axis	Proliferation and anti-apoptosis	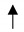	Zehendner CM et al., Am. J. Respir. Crit. Care Med. 2020 [179]
LnRPT	PDGF-BB-induced hyperproliferation of rat PASMCs	Notch signaling pathway	Proliferation	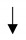	Chen J et al., Am. J. Respir. Cell Mol. Biol. 2018 [180]
SMILR	Human PASMCs, MCT-induced PH in Rats	RhoA/ROCK/ miR-141signaling	Vascular remodeling and PAH	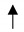	Lei S et al., Am. J. Physiol. Heart Circ. Physiol. 2020 [181]
MANTIS	MCT-induced PH rat models	BRG1	Angiogenesis and apoptosis	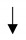	Leisegang MS et al., Circulation 2017 [182]
CASC2	PASMCs Hypoxic PAH in rats	α-SMA	Inhibits proliferation and migration	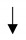	Gong J et al., Respir. Res. 2019 [183]

Abbreviations: PAH, pulmonary arterial hypertension; PASMCs, pulmonary artery smooth muscle cells; PAECs, pulmonary arterial endothelial cells; MCT, monocrotaline; MEG3, maternally expressed 3; TYKRIL, tyrosine kinase receptor inducing IncRNA; LnRPT, lncRNA regulated by PDGF and transforming growth factor β; PDGF, platelet-derived growth factor; SMILR, smooth muscle-enriched long noncoding RNA; CASC2, cancer susceptibility candidate 2; PTEN, phosphatase and tensin homolog; IGF1R, insulin-like growth factor 1 receptor; NF-κB, nuclear factor kappa B; PDGFR, platelet-derived growth factor receptor; ROCK, rho-associated protein kinase; BRG1, brahma-related gene 1; α-SMA, alpha-smooth muscle actin.

## 5. Microfluidics for Epigenetic Analysis

Epigenetics studies on molecular mechanisms that change gene activity and expression while leaving out the alteration in DNA sequence [184]. Epigenetic modification, e.g DNA methylation and histone alteration, not only play essential parts in gene expression and regulation, but also are important in cellular processes, for example stem cell pluripotency/differentiation and tumorigenesis. The technique that probes the in vivo interactions of DNA-proteins is called chromatin immunoprecipitation (ChIP), which is a prominent tool for investigating epigenetic mechanism. Nevertheless, the conventional ChIP assays have the drawback of requirement millions of cells per test and hence are not feasible for analysis of samples from patients and laboratory animals. Micro-total analysis systems (MicroTAS) or also called Lab-on-a-Chip technology use microfluidics and microfabrication technology to minimize the analytical volume, and cost of chemical consumptions hence allow high throughput sensing and analyzing [185,186]. The LoC technology opens up the possibility for development and performing microfluidic ChIP assays [187]. 

Wu and co-authors reported a microfluidic device to perform sensitive, rapid, automated ChIP analysis with low number of cells (2000 cells) while the specificity of the assays was still remained [188]. Cao and Lu reported a simple microfluidic device that integrates sonication and immunoprecipitation (IP) for epigenetic assays. These devices offered highly sensitive tests with 100 cross-linked cells for ChIP or 500 pg of genomic DNA for methylated-DNA IP. The process on chip took for only 1 h [189].

Epigenetic assays are emerging as an important tool for the early diagnosis of cancer. In this application, hyper-methylated DNA (hm-DNA) is critical for epigenetic based assays. De and co-authors [190] reported a microfluidic lab-on-a-chip platform to capture hm-DNA for less than 5 min with an efficiency of 71% to 92% with elution volume of 25 µL and 100 µL respectively. The authors utilized microfluidic solid phase extraction (µSPE) for hm-DNA, to capture, enrich and purify hm-DNA, which resulted in an enrichment factor of 28 times. 

DNA methylation is one of the major epigenetic modifications. The current development of micro, nanotechnology and advanced material science gives rise to a rapidly increased application of microfluidics and point-of-care devices in diagnosis, especially in early-stage cancer detection using DNA-methylated-based technologies [191]. The aim of the researchers in the fields can be bringing these point of care devices to the market to serve not only developed countries but also low-income regions [185,186,191,192].

## 6. Conclusions and Perspectives

Despite considerable progress in new drug target development and the emergence of current vasodilator therapies, PAH remains a high-mortality disease. It is a progressive disease with sex disparity affecting the entire pulmonary and cardiovascular systems. A microfluidic chip model mimicking the PAH-afflicted artery could mimic the hormone response in a similar fashion to that observed in PAH patients. When growing cells on this device, cells from male patients develop a more severe form of the disease compared to cells from female patients. This study suggests that the newly developed PAH tissue chip model responds to hormones in a similar fashion to that observed in PAH patients, and that it is a useful model for studying not only the mechanism of sex disparity to develop sex-specific therapies for patients with PAH, but also epigenetic modulators such as sirtuin activators to develop new targets for PAH therapy.

DNA methylation, histone post-translational modifications (acetylation, methylation), and ncRNAs have been implicated in multiple facets of PAH, including regulation of abnormal proliferation, apoptosis resistance, inflammation, and fibrosis, as well as vascular and RV remodeling and mitochondrial dysfunction. PASMC and endothelial cell proliferation, migration, and survival are directly regulated by epigenetic modifications. Although some epigenetic mechanisms need to be further elucidated, strong supporting data suggest that epigenetic modulators could be very promising and emerging targets for PAH therapy. However, further studies need to be performed in detail for confirmation of the effects before testing these epigenetic modifiers in clinical trials. Multiple studies have shown dysregulation of miRNAs in the pathogenesis of PAH. However, the application of miRNAs might be limited because of their degradation before modulating the targets. This drawback of miRNAs in therapies can be compromised by using chemical modifications, modified delivery systems, or pharmaceutic formulations such as engineered nanoparticles to improve miRNA stability.

## Figures and Tables

**Table 1:** List of sirtuin modulators in PAH and RVH.

Sirtuin Members/Molecular Pathway	Experimental Animal Model	Modulators	Summary	References and Year
Sirt1 (Sirt1 activator)	MCT, rat	Resveratrol	Resveratrol inhibits RV remodeling and dysregulation in MCT-induced PAH.	Vazquez-Garza E et al., Oxid Med Cell Longev. 2020 [151]
SphK1/S1P signaling	MCT, rat	Resveratrol	Resveratrol improves pulmonary vascular remodeling and attenuates the PAH development by inhibiting SphK1/S1P-mediated NF-kappaB activation and subsequent cyclin D1 expression.	Shi W et al., Life Sci. 2018 [156]
Sirt1	Human, rat	Resveratrol, SRT1720	Resveratrol and SRT1720 alleviate RVSP and RVH, significantly reduce PASMC proliferation, and promote PASMC apoptosis via mediating Sirt1.	Yu L et al., Cell Physiol Biochem. 2017 [155]
	Rat	Resveratrol	Resveratrol is not responsible for attenuation of RV remodeling caused by MCT; however, it inhibits PASMC hypertrophy in the pulmonary vessels.	Wilson DN et al., Pathophysiology. 2016 [157]
Sirt1, eNOS	MCT, rat, hypoxia, CR		SIRT1 induction inhibits induced PAH in experimental animal models by targeting eNOS pathway.	Ding M et al., J Cardiovasc Pharmacol. 2015 [141]
Arginase II, PI3K/Akt signaling pathway	Neonatal rat model of chronic hypoxia-induced pulmonary hypertension	Resveratrol	Resveratrol normalizes RV hypertrophy and pulmonary artery remodeling by inhibiting hypoxia-induced arginase II expression mediated via the PI3K/Akt signaling pathway.	Chen B et al., Am J Physiol Lung Cell Mol Physiol. 2014 [150]
Sirt1, atrophic ubiquitin ligases atrogin-1	MCT, rat	Resveratrol	Resveratrol ameliorates medial thickening of intrapulmonary arteries and phenotypes of pulmonary hypertension induced by MCT, such as RVSP and RVH.	Paffett ML et al., Vascul Pharmacol. 2012 [153]
Sirt1	MCT, rat	Resveratrol	Resveratrol attenuates hypertrophy of right-ventricle heart.	Yang DL et al., Clin Exp Pharmacol Physiol 2010 [158]
Sirtuins	Human, bovine, MCT-rat	Resveratrol	Resveratrol treatment ameliorates RV systolic pressure and pulmonary arterial remodeling by exerting anti-inflammation, antioxidant, and antiproliferation effects in the pulmonary arteries.	Csiszar A et al., Hypertension 2009 [152]
Sirt1, PGC-1alpha, and its downstream effectors	Human, rat, chronic hypoxia	Stac-3	Sirt1 inhibition exacerbates remodeling of pulmonary vessels. SIRT1 upregulation inhibits PASMC proliferation.	Zurlo G et al., J Hypertension 2008 [140]
Sirt3	Human, mice	Angiotensin II	Downregulation of Sirt3 inactivates SOD2 causing PH. Hypertension is significantly increased in Sirt3KO mice responding to angiotensin II.	Dikalova AE et al., Circulation research 2017 [159]
Sirt3	Human, rat		Sirt3 is downregulated in PAH, and its induction reverses PAH phenotype. Sirtuin-3 loss-of-function SNP rs11246020 is correlated with clinical IPAH.	Paulin R et al., Cell Metab. 2014 [144]
Sirt4/PDH/GDH	Human, mouse, rat	Bone marrow-derived exosomes	SIRT4 expression is increased in prolonged hypoxia. A decrease in Sirt4 expression is correlated with enhanced expression of PDH and GDH, and mitochondrial dysfunction of PAH was reversed by bone marrow-derived exosomes.	Hogan S E et al., J Physiol Lung Cell Mol Physiol. 2019 [147]

Abbreviations: PAH, pulmonary arterial hypertension; IPAH, idiopathic pulmonary arterial hypertension; MCT, monocrotaline; eNOS, endothelial nitric oxide synthase; PASMC, pulmonary artery smooth muscle cells; CR, calorie restriction; PDH, pyruvate dehydrogenase; GDH, glutamate dehydrogenase; Sirt1, Sirtuin-1; Sirt3, Sirtuin-3; Sirt4, Sirtuin-4; Sphk1, sphingosine kinase 1; SP1, specificity protein 1; PI3K/Akt, phosphoinositide 3-kinase/protein kinase B; PGC-1alpha, peroxisome proliferator-activated receptor-gamma coactivator (PGC)-1alpha; NF-κB, nuclear factor kappa B; SNP, single-nucleotide polymorphism; RV, right ventricle; RVSP, RV systolic pressure; Stac-3, Sirt1 activator agent.

## Data Availability

Not applicable.

## Abbreviations

PH	pulmonary hypertension
PA	pulmonary artery
PAH	pulmonary arterial hypertension
PVR	pulmonary vascular resistance
RV	right ventricular
RHF	right-heart failure
RVH	right-ventricular hypertrophy
mPAP	mean pulmonary artery pressure
LVEDP	left-ventricular end-diastolic pressure
WU	Wood units
WHO	World Health Organization
COPD	chronic obstructive pulmonary disease
IPAH	idiopathic PAH
HPAH	heritable PAH
APAH	associated PAH
PASMCs	pulmonary arterial smooth muscle cells
PAECs	pulmonary arterial endothelial cells
PAADCs	pulmonary arterial adventitial cells
HDMs	histone demethylases
HDACs	histone deacetylases
Sirt1	Sirtuin-1
Sirt3	Sirtuin-3
Sirt4	Sirtuin-4
BRD4	bromodomain-containing protein 4
5-HT	5-hydroxytryptamine
ERAs	endothelin receptor antagonists
PCAs	prostacyclin analogues
PDE-Is	phosphodiesterase 5 inhibitors
sGCs	soluble guanylate cyclase stimulators
FDA	the US Food and Drug Administration
CCBs	calcium channel blockers
NO	nitric oxide
cGMP	cyclic guanosine monophosphate
sGC	soluble guanylyl cyclase
PDE5	phosphodiesterase-5
cGMP	cyclic guanosine monophosphate
GMP	guanosine monophosphate
PGI2	prostacyclin
cAMP	cyclic adenosine monophosphate
PKA	protein kinase A,
SC	subcutaneous
IV	infusion
ECE	endothelin-converting enzyme
IP3	inositol triphosphate
E2	estradiol
DHEA-S	dehydroepiandrosterone sulfate
Treg	regulatory T cells
Erα	estrogen receptor alpha
Erβ	estrogen receptor beta
TFAM	mitochondrial transcription factors
NFAT	nuclear factor of activated T-cells
NLPR3FSP-1	NLR (NOD like receptor) family pyrin domain-containing 3 fibroblast-specific protein 1
CD31	platelet endothelial cell adhesion molecule (PECAM-1) also known as cluster of differentiation 31
CYP1B1	cytochrome P450 family 1 subfamily B member 1
ncRNAs	noncoding RNAs
lncRNAs	long noncoding RNAs
circRNAs	circular RNAs
CpG	cytosine-phosphate-guanine
DNMTs	DNA methyltransferases
MTase	methyltransferase
H3K3me3	histone 3 lysine 4 trimethylation
H3K27me3	histone 3 lysine 27 trimethylation
K4me3	lysine 4 trimethylation
MeCP1	methylated CpG-binding protein 1
MeCP2	Methylated CpG-binding protein 2
MBDs	methyl-CpG-binding domain proteins
SOD2	superoxide dismutase 2
FHR	Fawn-hooded rats
HIF-1α	hypoxia-inducible factor
Kv1.5	voltage-gated K^+^ channels
siRNA	small interfering RNA
BMPR2	bone morphogenetic protein receptor 2
TBX4	T-box 4
ACVRL1	activin A receptor-like type 1
ENG	endoglin
SMAD9	small mothers against decapentaplegic homolog 9
CAV1	caveolin-1
KCNK3	potassium channel subfamily K member 3
ATP13A3	ATPase 13A3
SOX17	SRY-box 17
AQP1	aquaporin 1
BMP9 (GDF2)	growth differentiation factor 2
EIF2AK4	eukaryotic translation initiation factor 2 alpha kinase 4
TGF-β	transforming growth factor beta
BMP/TGF	bone morphogenic protein/transforming growth factor
WES	whole genome sequencing
SPS	small-patella syndrome
HMTs	histone methyltransferases
JmjC	Jumonji C family
LSD	lysine-specific demethylases
FAD	flavin adenine dinucleotide
α-KG	α-ketoglutarate
CAM	cell adhesion molecules
ICAMs	intercellular adhesion molecules
VCAMs	vascular cell adhesion molecules
MRTF-A	myocardin-related transcription factor A
NF-κB	nuclear factor kappa B
ASH2	small homeotic 2
WDR5	WD repeat domain 5
EZH2	enhancer of zeste homolog 2
TAC	transverse aortic constriction
ROS	reactive oxygen species
SOD1	superoxide *d*ismutase type 1
GSK-J4	a KDM6B inhibitor
LSD1	lysine-specific histone demethylase 1A
HATs	histone acetyltransferases
HDACs	histone deacetylases
BRDPs	bromodomain-containing proteins
eNOS	endothelial nitric oxide synthase
PPHN	persistent PH of the newborn
PAB	pulmonary artery banding
MCT	monocrotaline
TSA	trichostatin A
VPA	valproic acid
MEF2	myocyte enhancer factor 2
IGF-1/pAKT	insulin-like growth factor-1/phosphorylation of protein kinase B
RVSP	RV systolic pressure
CO	cardiac output
TPR	pulmonary vascular resistance
BRD	bromodomain
BET	bromodomain and extra-terminal domain
BCL2	B-cell lymphoma 2
FOXM1	Forkhead Box M1
NAD+	nicotinamide adenine dinucleotide
FOXO1	Forkhead box protein O1
CR	calorie restriction
MnSOD	manganese superoxide dismutase
SOD2	superoxide dismutase 2
Cat	catalase
Sirt3KO	Sirtuin-3 knockout
PDK	pyruvate dehydrogenase kinase
PDH	pyruvate dehydrogenase complex
GDH	glutamate dehydrogenase
DCA	dichloroacetate
UCP2	uncoupling protein 2
PI3K/AKT	phosphoinositide 3-kinase/protein kinase B
Sphk1	sphingosine kinase 1
SP1	specificity protein 1
Pri-miRNAs	primary miRNAs
RISC	RNA-induced silencing complex
